# How to Apply Plaster of Paris

**Published:** 1899-01

**Authors:** Lester Keller

**Affiliations:** Ironton, O.


### HOW TO APPLY PLASTER OF PARIS.

By LESTER KELLER, M.D., IRONTON, O.

The surgeon who finds plaster of paris satisfactory for a dressing is the one who knows how to apply it. I believe the reverse of this proposition is equally true. Some plain instructions will, I think, overcome some of the difficulties in its application. I will not attempt to say where it should be used, but the same directions will help in all cases where it is necessary.

The greatest objections are that it will not set quick, and that it will crumble. Let us prepare bandages. We want some cheesecloth, fresh plaster, a pepper-box with large perforations, full of pulverized alum, and a smooth board about 10 inches wide and 3 feet long. Tear your cloth three times as wide as you want your bandages and from 4 feet to 6 feet long. Spread it out on the board, and as an assistant pours on the dry plaster rub it in well with the hands so as to fill up all the meshes of the cloth. Have a little alum dusted on, fold the cloth on itself, so as to make three thicknesses, roll up very loosely. Treat the entire roll that way, and remember a little excess of plaster is a very good thing. When we are ready to put on the dressing we want plenty of rolls so prepared, two bowls of warm water, a piece of oilcloth to put under the dish, a large sugar-shaker full of dry plaster, a big apron, and an assistant.

Put one of the rolls in the water, and after it has become wet through, give it a gentle squeeze and apply it to the limb. After it is on, dust on some dry plaster from the sugar-shaker, dip your hands in the water and rub them over the outside of the bandage. Apply rolls as long as necessary to get the bandage heavy enough, and then give it a good coat of plaster on the outside. Your assistant will be kept busy keeping the bandage wet (only one should be wet at a time), dusting on the plaster, and occasionally giving a dash of alum. Keep your hands wet rubbing them over the surface of the dressing. Make your strips of cloth short so that the inside of the roll will not set before you get it all on. Work your plaster wet and it will not crumble. Put in the alum to make it set quickly. Above all, work quickly.—*Medical Council.*

